# INFLUENCE OF COGNITION AND FUNCTIONAL LIMITATIONS ON HOSPITALIZATIONS AMONG OLDER ADULTS IN THE UNITED STATES

**DOI:** 10.1093/geroni/igad104.3128

**Published:** 2023-12-21

**Authors:** Augustine Boateng, Ari Friedman

**Affiliations:** University of Pennsylvania, Philadelphia, Pennsylvania, United States; University of Pennsylvania, Philadelphia, Pennsylvania, United States

## Abstract

**Background:**

Older adults frequently seek emergency department care, with 45 visits for every 100 older adults. Many health events in later stages of life can be attributed to health conditions and behaviors during middle-age. Yet, while qualitative and quantitative analyses exist, documenting the reasons for emergency department visits among all comers, no study focuses specifically on the determinants of hospitalization among middle-aged and older adults using the emergency department. Additionally, the impact on longer-term outcomes starting in mid-life remains unknown.

**Objectives:**

To determine whether impaired cognition, functional limitations, and multimorbidity in midlife predict subsequent hospitalizations in older age among older adults in the United States using core gerontology concepts such as functional limitations, multimorbidity, and cognitive impairment.

**Methods:**

We performed secondary analysis of 2,221 participants from the Midlife in the United States (MIDUS) Series, a longitudinal study involving US adults aged 45 to 75 years using a balanced Generalized Estimation Equation (GEE) approach. Covariates such as as gender, education, marital status, income, race/ethnicity, social stressors, and social connectedness were adjusted for.

**Results:**

Findings suggests that impaired cognition (p< 0.05), having two or more health conditions (p< 0.001) and functional limitations (p< 0.05) in midlife increases the number of hospitalizations in old age a decade later.

**Conclusions and Implications:**

Health behaviors and conditions in midlife can significantly impact the number of hospitalizations among older adults and increase costs associated with hospitalizations. The promotion of preventive care, targeted interventions, and the adoption of healthier lifestyle habits in midlife may reduce hospital admissions in old age.

